# Improving protein fold recognition by random forest

**DOI:** 10.1186/1471-2105-15-S11-S14

**Published:** 2014-10-21

**Authors:** Taeho Jo, Jianlin Cheng

**Affiliations:** 1 Department of Computer Science, Informatics Institute, C. Bond Life Science Center, University of Missouri, Columbia, MO 65211, USA

## Abstract

**Background:**

Recognizing the correct structural fold among known template protein structures for a target protein (i.e. fold recognition) is essential for template-based protein structure modeling. Since the fold recognition problem can be defined as a binary classification problem of predicting whether or not the unknown fold of a target protein is similar to an already known template protein structure in a library, machine learning methods have been effectively applied to tackle this problem. In our work, we developed RF-Fold that uses random forest - one of the most powerful and scalable machine learning classification methods - to recognize protein folds.

**Results:**

RF-Fold consists of hundreds of decision trees that can be trained efficiently on very large datasets to make accurate predictions on a highly imbalanced dataset. We evaluated RF-Fold on the standard Lindahl's benchmark dataset comprised of 976 × 975 target-template protein pairs through cross-validation. Compared with 17 different fold recognition methods, the performance of RF-Fold is generally comparable to the best performance in fold recognition of different difficulty ranging from the easiest family level, the medium-hard superfamily level, and to the hardest fold level. Based on the top-one template protein ranked by RF-Fold, the correct recognition rate is 84.5%, 63.4%, and 40.8% at family, superfamily, and fold levels, respectively. Based on the top-five template protein folds ranked by RF-Fold, the correct recognition rate increases to 91.5%, 79.3% and 58.3% at family, superfamily, and fold levels.

**Conclusions:**

The good performance achieved by the RF-Fold demonstrates the random forest's effectiveness for protein fold recognition.

## Background

Proteins are the fundamental functional units in living systems. Protein tertiary (three-dimensional) structures at the molecular level are necessary to understand the functions of proteins. However, due to the significant cost of experimentally determining the tertiary structures of proteins, the number of known 3D protein structures is about 200 times smaller than the number of known protein sequences [1,2]. Therefore, it is important to develop computational methods to predict protein structures from protein sequences [3]. Recognizing a known structure that is similar to the unknown structure (i.e. fold recognition) is an important step of the template-based protein structure modeling approach that uses the known structure as a template to construct a structural model for the target protein [4,5].

Since the number of unique protein structures appears to be limited (e.g., several thousand) according to the structural analysis on all the tertiary protein structures in the Protein Data Bank (PDB) [6], it is possible to identify one correct template structure (fold) for a large portion of target proteins. This is particularly the case if a target protein has a significant sequence identity with one of template proteins with a known tertiary structure. Fold recognition becomes very challenging when the sequence identity of the target protein and template proteins is low, i.e., in the twilight zone. Numerous research endeavors have been devoted to developing sensitive methods to improve fold recognition in the twilight zone. Machine learning methods have been used to tackle the problem effectively by casting the fold recognition as a binary classification problem to decide whether or not a target protein shares the same structural fold with a template protein in a protein structure library [6-8].

Given a number of features describing the pairwise similarity between two proteins (e.g., a target protein and a template protein), the objective of the classification is to predict if the two proteins share a similar tertiary structure (fold). The problem can often be divided into three difficulty levels that range from the easiest family level (i.e. two protein belonging to the same family), to the superfamily level, and to the hardest fold level. This roughly corresponds to the decrease in sequence identity between two proteins. Proteins sharing similar structures have a relatively high sequence similarity if they are in the same family, moderate or little sequence similarity if in the same superfamily, and almost no sequence similarity if in the same fold.

Random forest is one of the most powerful machine learning methods known for its good interpretability and its efficiency in handling very large training datasets [9]. Random forest grows a large number of decision trees based on a subset of randomly selected features and a fraction of randomly selected training data points. All the trained trees are applied to a new data point to make prediction. The majority vote of the ensemble of trained decision trees is used as the final prediction for the data point. The average decision based on a large number of decision trees makes random forest robust against noisy data, irrelevant features, and unbalanced class distribution. Random forest has delivered an excellent performance in broad classification tasks that compares favorably with other ensemble classifiers such as Adaboost [10], and its performance is generally comparable to other state-of-the-art classifiers such as Support Vector Machine (SVM) as well [11]. Random forest has been used extensively in a wide variety of domains [12-14] including protein fold classification [15-17], which is related to, but different than protein fold recognition. The fold recognition problem addressed in this paper is to recognize proteins that have similar tertiary structures to target proteins, while the protein classification [15,16], and [17] is to classify a single protein sequence into a number of structural folds. On contrary, we applied random forest to classify if a pair of proteins (one target protein and one template protein) shares the same structure. The classification scores are then used to rank template proteins based on their structural relevance (i.e. the classification score) with a target protein. Many methods have been developed to improve the accuracy of recognizing structurally similar folds when there is little sequence similarity between a target and a template protein, such as PSI-BLAST [18], HMMER [19], SAM-T98 [20], SSHMM [21],THREADER [22], FUGUE [23], SPARKS [24], SP3 [25], HHpred [26], FOLDpro [5], SP4 [27], SP5 [28], RAPTOR [29], SPARKS-X [30], and BoostThreader [31].

In this work, we applied the random forest method (i.e. RF-Fold) to address the fold recognition problem and evaluated its performance on the standard Lindahl's dataset [32], on which many previously established methods had been benchmarked. In comparison with 17 existing methods, RF-Fold's performance was comparable to that of the state-of-the art methods, demonstrating the effectiveness of the random forest method in protein fold recognition.

## Methods

### Random forest method for protein fold recognition

The decision tree method for classification had been widely used in many domains due to its simplicity and good interpretability after Leo Breiman et al. introduced it in 1984 [33]. However, the accuracy of a single decision tree is often lower than more advanced classification methods such as support vector machines or neural networks, which limits its application in accuracy-critical domains. The more recent development of the decision tree methodology found that using an ensemble of decision trees constructed from randomly selected features and training data not only often yielded significantly higher accuracy than a single decision [34,35], but also often surpassed the accuracy of other most advanced machine learning methods. This new approach is called random forest. Random forest is a meta-learning algorithm for classification, which consists of a bag of separately trained decision trees. Therefore, it inherits the advantages of decision tree methods such as easy training, fast prediction, and good interpretability. Because random forest selects a random subset of input features to construct each decision tree, the average prediction of a sufficient number of decision trees is robust against the existence of irrelevant features, which partially contributes to its good accuracy. Furthermore, the random selection of a subset of training data to train each tree also leads to an ensemble of decision trees that are resistant to noise and disproportional class distribution in the training data.

**Figure 1:**
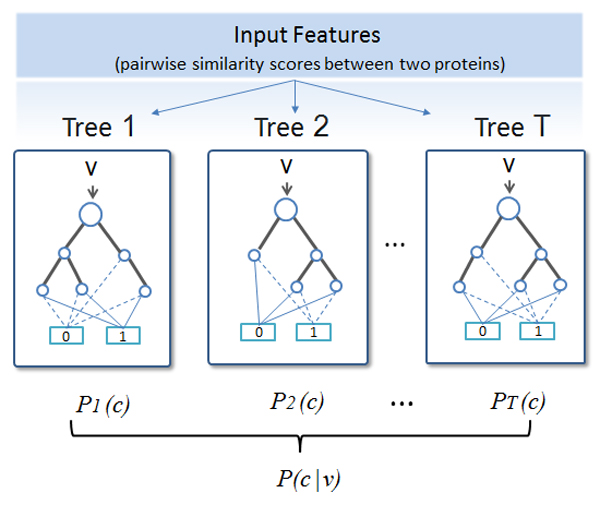
**A random forest to classify if two proteins share the same fold**. The random forest is comprised of T decision trees. Each tree predicts the probability of class c conditioned on an input feature vector (v) representing the similarity between two proteins. Class c is either 0 (not in the same fold) or 1 (in the same fold). The average probability of all the trees is calculated as P(c|v). The class c with the higher value is the predicted class for the input feature vector.

In our study, each decision tree in the random forest was trained to predict if two proteins share a similar structural fold or not from a list of input features describing similarity between the two proteins (see Section 2.2 for the description of the training data and features). A number of features used to construct each decision tree were randomly selected from the total 84 features. The random forest method was implemented by using the randomForest R package [37]. The decision trees were trained by the standard decision tree training algorithm that maximized the information gain in selecting a feature to partition the training data. After training, each tree (T) was able to predict the probability of each class (1: in the same fold or 0: not in the same fold) given an input feature vector representing a protein pair (a target protein and a template protein). The average probability predicted by these trees was calculated and the class with higher predicted probability was the prediction. Figure 1 illustrates how the random forest makes a prediction. The trained random forest is used to predict if a target protein has the similar fold with each template protein in the test data set through cross-validation. The top one or five templates with the higher predicted probability to share a fold with the target protein were obtained for evaluation.

### Data set and features

We trained and tested RF-Fold on the FOLDpro dataset [5]. The FOLDpro dataset used the proteins in Lindahl's benchmark dataset [32] derived from the SCOP [7] database (version 1.39). The Lindahl's dataset includes 976 proteins, among which 555 proteins have at least one positive match with other proteins at the family level, 434 proteins at the super family level, and 321 proteins at the fold level. The pairwise sequence identity of any pair in the dataset is <= 40%. In the FOLDpro dataset, 84 features were extracted for each of all 976 × 975 distinct protein pairs in order to classify if a pair of proteins (one target / query protein and one template protein) share the same structure at the family, superfamily, or fold level. The features were extracted using existing, general-purpose alignment tools as well as protein structure prediction programs in five categories, including sequence/family information, sequence-sequence alignment, sequence-profile alignment, profile-profile alignment, and structural information. For the features of sequence/family information, the compositions of a single amino acid (monomer) and an ordered pair of amino acid (dimer) were computed and transformed into similarity scores using the cosine, correlation, and Gaussian kernel functions. For sequence-sequence alignment features, PALIGN [38] and CLUSTALW [39] were used to extract pairwise features associated with sequence alignment scores of a pair of proteins. For sequence-profile alignment features, PSI-BLAST, HMMER-hhmsearch [40] and IMPALA [41] were used to extract profile-sequence alignment features between the target profile and the template sequence. For profile-profile alignment features, five profile-profile alignment tools CLUSTALW, COACH of LOBSTER [42], COMPASS [43], HHSearch [44] and PRC (Profile Compiled, http://supfam.org/PRC) were used to align target and template profiles to obtain profile-profile alignment scores. For structural features, based on the global profile-profile alignments obtained with LOBSTER, structural features of query proteins predicted using the SCRATCH suite [45-49] were compared with that of template proteins to obtain structural compatibility scores.

The small portion of pairs belonging to the same protein family, superfamily, or fold was labelled as positive examples because they shared the same structural folds. The vast majority of protein pairs that did not have structural similarities were labelled as negative examples.

### Training and benchmarking

We divided all protein pairs into 10 equal-size subsets for 10-fold cross validation purposes. We put all the target-template pairs associated with the same target protein into the same subset. Nine subsets were used for training and the remaining subset was used for validation. We removed all the pairs in the training dataset that used targets in the test dataset as templates. This procedure was repeated 10 times and the sensitivity and specificity of fold recognition were computed across the 10 trials. We also compared RF-Fold with 17 other methods by fold recognition rates for top-one ranked templates and for top-five ranked templates as in [5,32]. Using the same evaluation procedure as in [5,29-32], we calculated the sensitivity by taking as predictions the top-one or the top-five template proteins ranked for each target protein by classification scores. Here the sensitivity was defined by the percentage of target proteins (with at least one possible hit) having at least one correct template ranked 1st, or within the top 5 [5,32].

## Results

### Comparison of random forest with a single decision tree

We compared the random forest consisting of 500 decision trees to a single decision tree in terms of the error rate (i.e. percent of incorrectly classified protein pairs). The error rate of the random forest classification was 0.566%, which was lower than 1.135% of a single tree (Table 1). It is worth noting that the error rate is very low because the dataset with only a small fraction of positive examples is highly imbalanced.

**Table 1:** Error rate of the random forest and a single decision tree on fold recognition dataset.

Method	Error rate (%)
Random forest	0.566
Single decision tree	1.135

### Effects of data imbalance on random forest

It is difficult to train a classifier on a highly imbalanced dataset in which one or more classes are extremely under-represented. The significant drawback of using training data with the imbalanced distribution of classes has been reported in [36]. The FOLDpro dataset is a very imbalanced dataset, which has 7,438 positive examples versus 944,162 negative examples. The ratio between the majority class and the minority class is 128:1. Training on such a dataset is difficult for most machine learning methods in general.

In order to assess how well the random forest approach handled imbalanced data, we trained the random forest classifier on 5 datasets, which had a ratio of negatives to positives of 128:1, 100:1, 75:1, 50:1, and 25:1. Table 2 shows how the numbers of correctly selected templates were at the family, superfamily, or fold level change with respect to the ratios in the10-fold cross validation. Except for the case with a 1:1 ratio, it appeared that the performance of random forest method was steady with different ratios of negative and positive examples.

**Table 2:** The number of correctly predicted template folds by random forest at the family level, superfamily level, and fold level under various ratios of negatives and positive training examples.

Ratio	Family		Superfamily		Fold	
			
	Top1	Top5	Top1	Top5	Top1	Top5
128:1 (Original)	469	508	275	344	131	187
100:1	457	505	271	335	119	183
75:1	466	503	273	330	118	181
50:1	459	504	276	329	120	174
25:1	452	504	270	324	114	181
1:1	190	287	81	157	49	107

### Effect of the number of features

**Figure 2:**
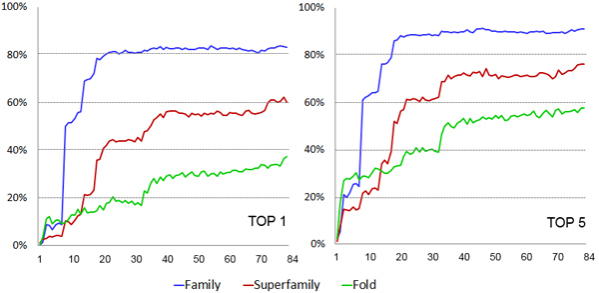
**The fold recognition sensitivities of the random forest with respect to different numbers of input features; Y-axis denotes the sensitivity and X-axis the number of features**.

The number of features used for training affects the performance of machine learning methods. We evaluated how the performance of the random forest changed with respect to the number of features used in training, which ranged from 1 to 84. Figure 2 shows the plots of the sensitivity of fold recognition of RF-Fold against the number of features at the family, super family, and fold levels for both top-one ranked templates and top-five ranked templates. The sensitivity for top-one (resp. top-five) ranked templates is defined as the percentage of target proteins having at least one correct template ranked no. 1 (resp. within the top-5) [32] by RF-Fold. The results showed that the performance of the random forest improved or stabilized as more features were used in training. However, it appeared to plateau out after 21 features at the family level, and after 41 features at the superfamily and fold level.

### Comparing RF-Fold with existing fold recognition methods

Table 3 shows the sensitivity of 18 fold recognition methods including RF-Fold at the family, superfamily, and fold levels, for the top-one and top-five predictions respectively. The sensitivity for top-one predictions (resp. for top-5 predictions) is defined as the percentage of target proteins having at least one correct template ranked by a method at top-one (resp. within top-five). The last two rows in this table show that RF-Fold performed better than FOLDpro [5] in all but one case in top-one at the family level, where the success rate of FOLDpro(85.0%) was similar to the RF-Fold(84.5%). At the superfamily level, the sensitivity of RF-Fold for the top-one or top-five predictions is 63.4% and 79.3%, about 9% higher than FOLDpro. RF-Fold had the largest improvement in top-one at the fold level, where its accuracy was 14.3% higher than FOLDPro's. The sensitivity of RF-Fold for the top-five predictions was 58.3%, which was 10% higher than FOLDpro.

**Table 3:** The sensitivity of 18 methods on the Lindahl's dataset.

Method	Family		Superfamily		Fold	
			
	Top1	Top5	Top1	Top5	Top1	Top5
PSI-Blast	71.2	72.3	27.4	27.9	4	4.7
HMMER	67.7	73.5	20.7	31.3	4.4	14.6
SAM-T98	70.1	75.4	28.3	38.9	3.4	18.7
BLASTLINK	74.6	78.9	29.3	40.6	6.9	16.5
SSERCH	68.6	75.5	20.7	32.5	5.6	15.6
SSHMM	63.1	71.7	18.4	31.6	6.9	24
THREADER	49.2	58.9	10.8	24.7	14.6	37.7
Fugue	82.2	85.8	41.9	53.2	12.5	26.8
SPARKS	81.6	88.1	52.5	69.1	28.7	47.7
SP3	81.6	86.8	55.3	67.7	30.8	47.4
HHpred	82.9	87.1	58	70	25.2	39.4
SP4	80.9	86.3	57.8	57.8	30.8	53.6
SP5	82.4	87.6	59.8	70	37.9	58.7
RAPTOR	**86.6**	89.3	56.3	69	38.2	58.7
SPARKS-X	84.1	90.3	59.0	76.3	**45.2**	**67.0**
BoostThreader	86.5	90.5	**66.1**	76.4	42.6	57.4
FOLDpro	85	89.9	55	70	26.5	48.3
RF-fold	84.5	**91.5**	63.4	**79.3**	40.8	58.3

The bold font denotes the highest sensitivity in their respective categories of prediction. The results of other methods are taken from [5,28,29]

RF-Fold performed better than most of methods in Table 3 and comparably to RAPTOR, SPARKS-X, and BoostThreader. Compared with RAPTOR, in most situations, RF-Fold shows some improvement of accuracy, while it performed worse than Raptor at top-1 family level and top-5 fold level. Compared with SPARKS-X, RF-Fold was less accurate at the fold level, but more accurate at the other two levels. Compared with BoostThreader, RF-Fold was less accurate in top-one at three levels, but more accurate in top-five at all three levels.

### Availability of RF-Fold software and source code

In order to facilitate the reuse and implementation of RF-Fold method, the online web service for fold recognition, the source code of the programs of random forest learning and classification, the scripts of generating pairwise features for a pair of proteins, the scripts of evaluating the fold recognition results, and the training and test datasets are released at http://calla.rnet.missouri.edu/rf-fold/. The readme .txt file describes how to train and test the random forest method for fold recognition (RF_learn and RF_classify programs), how to evaluate the performance on the benchmark data set (Calculate-lindahl-Top1-Top5.sh), the datasets used to do cross-validation, and the scripts used to generate 84 pairwise features for a pair of proteins (32 Perl scripts in scripts_feature_generation sub-directory). Based on the document and programs, any user can create his/her own training and test datasets and train / test his/her own random forest classifier for protein fold recognition from scratch. The software, source code and data are released under the GNU General Public License. Anyone can freely reuse the software and source code for any purpose (e.g., protein fold recognition, homology detection, and protein tertiary structure prediction). Any technical problems may be addressed to the email box of the corresponding authors. Based on users' feedback, additional documents, utility programs, test examples, and data will be added in order to facilitate the development of random forest methods for protein fold recognition.

## Conclusions

In this study, we developed a random forest method (RF-Fold) to recognize protein folds. The method was systematically validated by varying the input features and the class distribution of training datasets on a standard fold recognition dataset. The random forest consisting of 500 decision trees yielded a low error rate than a single decision tree on a highly imbalanced dataset. The random forest also delivered a good, steady performance regardless of the different ratios of negative and positive examples. Compared with 17 other different fold recognition methods, the performance of the RF-Fold is generally comparable to the best performance. The results achieved by the RF-Fold demonstrated the effectiveness of using the random forest algorithm in protein fold recognition. In the future, we plan to further evaluate the performance of RF-Fold on a standard protein homology detection dataset [50], independent CASP datasets [51], and to build a protein tertiary structure prediction web server based on RF-Fold for the community to use. Furthermore, the sensitivity of RF-Fold for the hardest fold recognition problem at the fold level is still relatively low (e.g. 40.8% for top-one predictions and 58.3% for top-five predictions), which is one of the major bottlenecks of template-based protein structure modeling. We will incorporate more informative features into RF-Fold to address this problem in the future.

## Competing interests

The authors declare that they have no competing interests.

## Authors' contributions

TJ implemented the algorithms and carried out the experiments. TJ and JC analyzed the data, wrote and edited the manuscript. TJ and JC approved the manuscript.

## Declarations

This article has been published as part of *BMC Bioinformatics *Volume 15 Supplement 11, 2014: Proceedings of the 11th Annual MCBIOS Conference. The full contents of the supplement are available online at http://www.biomedcentral.com/bmcbioinformatics/supplements/15/S11.
